# Pharmacological Evaluation and Preparation of Nonsteroidal Anti-Inflammatory Drugs Containing an *N*-Acyl Hydrazone Subunit

**DOI:** 10.3390/ijms15045821

**Published:** 2014-04-04

**Authors:** Thais Regina Ferreira de Melo, Rafael Consolin Chelucci, Maria Elisa Lopes Pires, Luiz Antonio Dutra, Karina Pereira Barbieri, Priscila Longhin Bosquesi, Gustavo Henrique Goulart Trossini, Man Chin Chung, Jean Leandro dos Santos

**Affiliations:** 1 School of Pharmaceutical Science, State University of São Paulo (UNESP), Rodovia Araraquara Jaú Km. 01, Araraquara, São Paulo 14801-902, Brazil; E-Mails: trfmelo@gmail.com (T.R.F.M.); rafaelchelucci@hotmail.com (R.C.C.); mariaelisalopes@yahoo.com.br (M.E.L.P.); luizdutra_qf@yahoo.com.br (L.A.D.); kakabarbieri85@yahoo.com.br (K.P.B.); bosquesi@fcfar.unesp.br (P.L.B.); chungmc@fcfar.unesp.br (M.C.C.); 2 Faculty of Pharmaceutical Science, University of São Paulo, Av. Professor Lineu Prestes 580, São Paulo 05508-900, SP, Brazil; E-Mail: gustavo.trossini@gmail.com

**Keywords:** anti-inflammatory, analgesic, hydrazone, molecular hybridization, non-steroidal anti-inflammatory, NSAID, docking, molecular modeling, COX

## Abstract

A series of anti-inflammatory derivatives containing an *N*-acyl hydrazone subunit (**4a**–**e**) were synthesized and characterized. Docking studies were performed that suggest that compounds **4a**–**e** bind to cyclooxygenase (COX)-1 and COX-2 isoforms, but with higher affinity for COX-2. The compounds display similar anti-inflammatory activities *in vivo*, although compound **4c** is the most effective compound for inhibiting rat paw edema, with a reduction in the extent of inflammation of 35.9% and 52.8% at 2 and 4 h, respectively. The anti-inflammatory activity of *N*-acyl hydrazone derivatives was inferior to their respective parent drugs, except for compound **4c** after 5 h. Ulcerogenic studies revealed that compounds **4a**–**e** are less gastrotoxic than the respective parent drug. Compounds **4b**–**e** demonstrated mucosal damage comparable to celecoxib. The *in vivo* analgesic activities of the compounds are higher than the respective parent drug for compounds **4a**–**b** and **4d**–**e**. Compound **4a** was more active than dipyrone in reducing acetic-acid-induced abdominal constrictions. Our results indicate that compounds **4a**–**e** are anti-inflammatory and analgesic compounds with reduced gastrotoxicity compared to their respective parent non-steroidal anti-inflammatory drugs.

## 1. Introduction

*N*-Acyl hydrazone (NAH), which is considered to be a privileged structure, has been used as the foundation for designing new analgesic and anti-inflammatory compounds, because the NAH subunit is a key pharmacophore for binding to and inhibiting cyclooxygenases (COX) [1]. Several mechanisms have been proposed to explain its recognition by COX. The first mechanism involves the relative acidity of the amide hydrogen moiety in NAH. The second mechanism involves the ability of the structure to stabilize free radicals that are structurally related to *bis*-allylic hydrigens in fatty acids, such as arachidonic acid (20:4) [2,3].

The long-term administration of nonsteroidal anti-inflammatory drugs (NSAIDs) is often limited by the emergence of gastrointestinal or cardiovascular complications [4]. It has been estimated that over two-thirds of regular NSAID users develop gastrointestinal complications [5]. The gastrotoxic effects of NSAIDs are caused by the inhibition of protective prostaglandin biosynthesis in the stomach and the direct contact between acidic drugs and mucosal cells [6]. Interestingly, some NSAIDs have been shown to be able to reduced ulcerogenic effects, which is attributed to the masking of the carboxylic acid moiety present in these drugs [6,7]. Therefore, anti-inflammatory and analgesic compounds with a NAH subunit are less likely to induce ulcers than NSAIDs without this subunit [8].

Several clinical studies investigating the cardiovascular toxicity of NSAIDs have shown that COX-2 and COX-1 inhibitors increase the risk of cardiovascular events, including myocardial infarction [9,10]. In fact, patients treated with high doses of diclofenac or ibuprofen have a high risk of vascular events, similar to patients treated with coxibs [11]. These deleterious effects of NSAIDs seem to be associated with the inhibition of prostacyclin synthesis, increased blood pressure, and oxidative stress-induced endothelial dysfunction [12].

**Figure 1.**
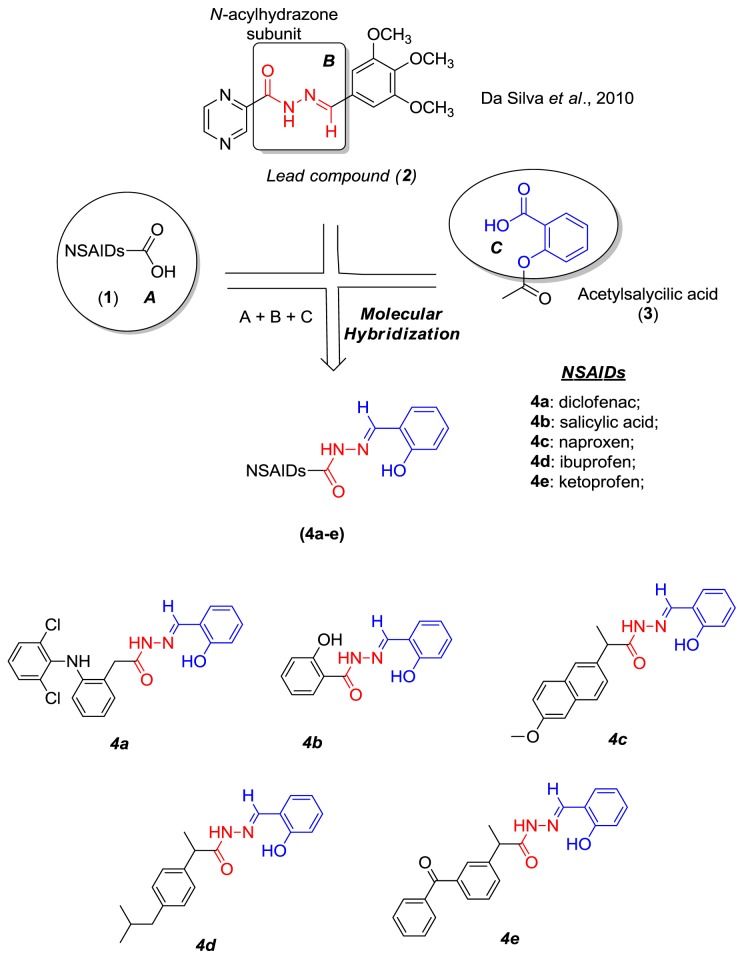
Molecular hybridization of NSAIDs with the NAH subunit (**4a**–**e**).

The antiplatelet and antithrombotic activities of compounds containing a NAH subunit have been extensively described [1,13–15]. Therefore, compounds with this subunit might exert cardioprotective effects. We have previously reported on the antiplatelet and antithrombotic activities of NSAIDs containing the NAH subunit (Figure 1) [16]. In that study, compounds **4a**–**e** reduced ADP-induced platelet aggregation by 18%–61.1%. Furthermore, *in vivo* experiments showed that compounds **4a** and **4e** prevented the incidence of thromboembolic events by 40% and 33%, respectively, whereas acetylsalicylic acid (ASA), which was used as the control drug, reduced the incidence of events by 30%. The bleeding time was also shorter in animals treated with these compounds than in animals treated with the same dose of ASA [16].

As part of our ongoing program to identify safe candidate drugs with anti-inflammatory properties, we describe the preparation, pharmacological evaluation, and docking studies of new NSAIDs containing an NAH subunit. The compounds were designed via a molecular hybridization approach using three different pharmacophore subunits, represented by their NSAID, ASA, and NAH structures (Figure 1). ASA was selected as a fragment, because it is the only NSAID suitable for primary and secondary cardiovascular prophylaxis [17–19]. Da Silva *et al.* described a series of compounds with a NAH subunit that had better analgesic and anti-inflammatory properties than celecoxib and indomethacin [20]. In particular, the most promising compound, (2-*N′*-[(*E*)-(3,4,5-trimethoxy-phenyl)methylidene]-2-pyrazinecarbohydrazide (2)) (Figure 1), was effective in a mouse model of chronic inflammation [20]. The three subunits were hybridized to generate compounds **4a**–**e**, which were designed as nongastrotoxic, anti-inflammatory, and analgesic compounds with cardioprotective effects.

## 2. Results and Discussion

### 2.1. Chemistry

**Figure 2.**
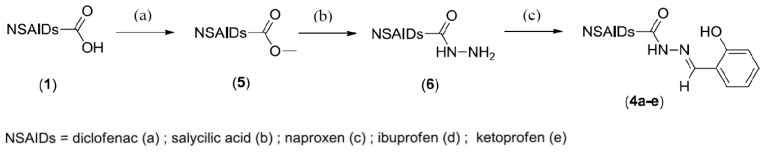
Preparation of compounds **4a**–**e**. Reagents and conditions: (a) CH_3_OH, H^+^, reflux (95%–99%); (b) NH_2_NH_2_ 64%, CH_3_OH, r.t. (90%–95%); (c) salycilic aldehyde, ethanol, H^+^, (61%–82%).

The synthetic routes used to prepare the anti-inflammatory derivatives (**4a**–**e**) are summarized in Figure 2. The first stage of the production of the NSAID derivatives involved two synthetic steps: (1) esterification of the carboxylic acid moiety of NSAIDs generated methyl ester derivatives with excellent yields (95%–99%); and (2) hydrazinolysis of the methyl esters (**5a**–**e**) generated the corresponding hydrazide derivatives (**6a**–**e**) with yields of 90%–95%. In the second stage, compounds **6a**–**e** were subjected to condensation reactions with salicylic aldehyde, resulting in the formation of a new series of NSAID-acylhydrazone derivatives (**4a**–**e**) with moderate to high yields (61%–82%).

The structures of the compounds were established by infrared spectroscopy, ^1^H-nuclear magnetic resonance (NMR) spectroscopy, mass spectrometry, and elemental analysis. The compounds were also subjected to high-performance liquid chromatography, which confirmed that the purity of each compound was >98.5%. The ^1^H-NMR spectra of the NSAID–NAH derivatives displayed one peak, corresponding to an ylidenic hydrogen, which was attributed to the *E*-diastereomer [20,21].

### 2.2. Anti-Inflammatory/Analgesic Activities and Ulcerogenicity Studies

The anti-inflammatory activity was evaluated using the rat carrageenan-induced paw edema model [22]. The results are expressed as percentage of inhibition (mean ± S.E.M) calculated according to the following equation:

(1) $$\text{Inhibition }(\%)=\{[{\left(V_{\text{t}}-V_{\text{o}}\right)}_{\text{control}}-{\left(V_{\text{t}}-V_{\text{o}}\right)}_{\text{treated}}]/{\left(V_{\text{t}}-V_{\text{o}}\right)}_{\text{control}}\}\times 100$$

where *V*_t_ and *V*_o_ relate to the average volume in the hind paw of the rats after given the compounds orally (treatment group) and before any treatment. Carrageenan-induced edema is a nonspecific inflammation induced by several inflammatory mediators. Although dose-response curves were constructed for each compound at doses of 10–400 μmol/kg bodyweight, only doses of ≥300 μmol/kg bodyweight inhibited paw edema, and the reductions in edema were similar at doses of 300 and 400 μmol/kg bodyweight. Therefore, the effects of compounds **4a**–**e**, the parent drugs (diclofenac, salicylic acid, naproxen, ibuprofen, and ketoprofen), and indomethacin were evaluated after an oral dose of 300 μmol/kg bodyweight. All the parent drugs were evaluated in the same way and their anti-inflammatory activities were similar to the reference drug, indomethacin.

As shown in Table 1, compounds **4b**–**e** exhibited low to moderate anti-inflammatory activities. Compound **4c** was the most active compound, with inhibitory rates of 35.9% and 52.8% at 2 and 4 h, respectively. All compounds (**4a**–**e**) were less active than indomethacin, except compound **4c** at 360 min. In addition, compounds **4a**–**b** and **4d**–**e** demonstrated weaker anti-inflammatory activities than the parent drugs. Compound **4a** did not inhibit carrageenan-induced paw edema. Compound **4c** was the only derivative with anti-inflammatory activity superior to the parent drug after 300 and 360 min.

Several studies have revealed that drug variants containing the NAH subunit show greater efficacy than the parent drug. For example, Effenberger *et al.* reported that a hybrid of doxorubicin containing a NAH subunit exhibited greater anticancer effects than unmodified doxorubicin and had a different mode of action [23]. Although our docking studies showed that the modified compounds interact with COX-1 and COX-2, the presence of the NAH subunit did not increase the anti-inflammatory activity of the compounds compared with the parent NSAIDs (Table 1).

The analgesic properties of compounds **4a**–**e** were evaluated using acetic-acid-induced abdominal constrictions in mice [24]. Dose–response curves were initially constructed for the doses of compound **4a**–**e**, the parent drugs, and dipyrone (the control drug) at doses of 10–400 μmol/kg bodyweight. However, the compounds only exhibited analgesic activity at doses of ≥100 μmol/kg bodyweight, and the effects were similar to doses of ≥100 μmol/kg bodyweight. Therefore, compounds **4a**–**e**, the parent drugs, and dipyrone were administered orally at doses of 100 μmol/kg bodyweight. The analgesic activities of the parent drugs were similar to that of dipyrone (Table 2).

Compound **4a** had the strongest analgesic effect and reduced abdominal constrictions by 42%. The analgesic effects of compounds **4b**–**c** were similar to control dipyrone at the same dose; however, compounds **4d**–**e** were weaker than dypirone. The analgesic effects of compounds **4a** and **4b** were greater than the parent drugs, while compounds **4c**–**e** showed comparable values to the parent NSAIDs. The introduction of the NAH subunit did not decrease the analgesic effect, but rather improved this activity compared to the parent drugs, as in the case of **4a**–**b**. Several reports have suggested that the NAH subunit contributes to the analgesic activity of the hybrid compound [1,2,20,25].

In the ulcerogenic study, the rats were orally administered the study drug at a dose of 300 μmol/kg bodyweight, as previously described [26,27]. The rats’ gastric mucosa was examined using a 4× binocular magnifier. The lesions were counted and divided into large (larger than 2 mm in diameter), small (1–2 mm) and punctiform (less than 1 mm). Interestingly, the number of ulcers was reduced in rats treated with compounds **4a**–**e** relative to rats treated with the parent drug (Table 3). For all of the parent drugs, the mean number of ulcers per rat was >57.8, and most of the ulcers were 1–2 mm or >2 mm in diameter. Celecoxib was used as a control drug and the corresponding results are also shown in Table 3. In rats treated with diclofenac, the mean number of lesions was 72 per rat after one dose and 5.7% of the lesions were >2 mm in diameter. The value of the mucosal damage index for diclofenac was 4, based on the presence of one large ulcer. The other parent drugs also scored 4 at the dose used (300 μmol/kg bodyweight). The COX-2 inhibitor celecoxib induced ulcers at a dose of 300 μmol/kg bodyweight and scored a damage index of 2 (Table 3).

Although the mean number of ulcers in rats treated with compound **4a** was lower than in rats treated with the parent drug, diclofenac (26 *vs.* 72 ulcers, respectively), the number of ulcers induced by compound **4a** was greater than the number induced by compounds **4b**–**e**. Nevertheless, the mucosal damage index for compounds **4a**–**e** was 2 based on the presence of 1–5 small ulcers. Over 80% of the lesions in rats treated with compounds **4a**–**e** had a punctiform appearance. Compound **4c** had the weakest ulcerogenic effect of compounds **4a**–**e**. The number of ulcers in rats treated with compound **4c** was 7.1, and most were punctiform lesions (91.5%). Compounds **4a**–**e** showed less ulcerogenic effects than the respective parent drug. Compounds **4b**–**e** induced mucosal damage comparable to celecoxib.

Several hybrid or prodrug NSAID derivatives with reduced gastro-ulcerogenicity compared with the parent drugs have been reported [6,27–30]. Chatterjee *et al.* suggested that gastro-ulceration could be reduced by masking the carboxylic acid moiety of NSAIDs [30]. The gastrotoxic effects of NSAIDs are mainly caused by the inhibition of protective prostaglandin biosynthesis in the stomach and direct contact between the acid drugs and the mucosal cells of the stomach [6,31].

Gastrointestinal bleeding, erosion, and ulcers were induced by a single therapeutic dose of NSAIDs, such as ketoprofen, in rats [32]. In humans, the administration of NSAIDs is the most common cause of gastroduodenal injury. Bleeding or perforation accounts for ≥1% of all the serious gastrointestinal complications associated with NSAIDs [33]. It has been estimated that around 16,000 people die every year as a consequence of NSAIDs, mainly attribuable to gastrointestinal complications [34]. Although COX-2 inhibitors are less gastrotoxic than COX-1 inhibitors, COX-2 inhibitors are associated with serious adverse cardiovascular events in some patients. Several clinical trials have been performed to determine the incidence of gastrointestinal complications during treatment with COX-2 inhibitors [35]. Therefore, NSAIDs with reduced gastro-ulcerogenicity are highly desirable for the long-term treatment of inflammatory conditions. In this study, we have shown that NSAIDs containing a NAH subunit exhibit weaker gastrotoxicity than the parent drugs via a mechanism that might involve masking the carboxylic acid moiety of the NSAIDs.

### 2.3. Docking Studies

We also performed docking studies to evaluate the interactions, affinities, and selectivities of compounds **4a**–**e** for COX-1 and COX-2. The crystallographic structures of COX-1 (pdb code 1 GB with a resolution of 3.4 Å) [36] and COX-2 (PDB code 1CX2 with a resolution of 3.0 Å) [37] were used as templates.

**Figure 3.**
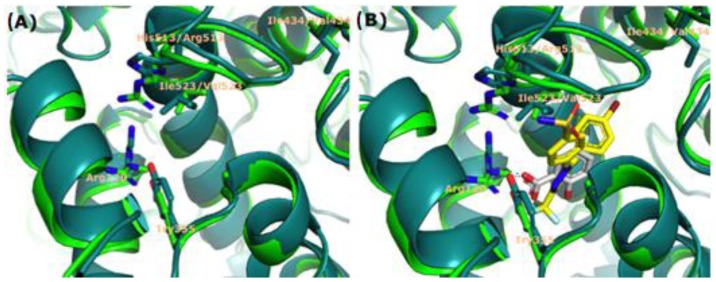
Crystallographic structures of COX-1 (1PTH; light green line) and COX-2 (1CX2; dark green line). (**A**) Major changes in isoforms 1 and 2 (Hist513Arg; Ile434Val and Ile523Val) and residues Arg120 and Try355, responsible for interaction with NSAIDs available in therapy; (**B**) Aligned structures of COX-1 and COX-2 bound to salicylic acid (white) or SC-558 (yellow), which interact with residues Arg120 and Try355, respectively.

We first performed detailed analyses of the active sites of COX-1 and COX-2 by aligning their crystallographic structures (PDB code: 1PTH/COX-1 and 1CX2/COX-2, respectively) using the PyMOL Molecular Graphics System, version 1.5.0.4 (Schrödinger, LLC, Portland, OR, USA). These analyses identified some differences in the residues that may affect the site selectivity of specific compounds to either isoform [38,39]. In particular, several residues, including Try355 and Arg120 (Figure 3A), allow the formation of hydrogen bonds, which favor interactions with COX-1 inhibitors [40,41]. One of the most widely reported difference is a change between isoleucine (COX-1) to valine (COX-2) at positions 434 and 523, respectively (Figure 3A). This modification increases the volume of the hydrophobic channel in the active site of COX-2 [36]. Another modification is the change from histidine to arginine at position 513 in COX-2 [42].

SC-558 is a selective COX-2 inhibitor that occupies a large volume within the active site. The sulfonamide moiety of SC-558 is located near the residue at position 523 (Ile in COX-1 and Val in COX-2). The larger volume of Ile relative to Val suggests that steric hindrance in this region is responsible for the greater selectivity of SC-558 for COX-2 [36].

**Figure 4.**
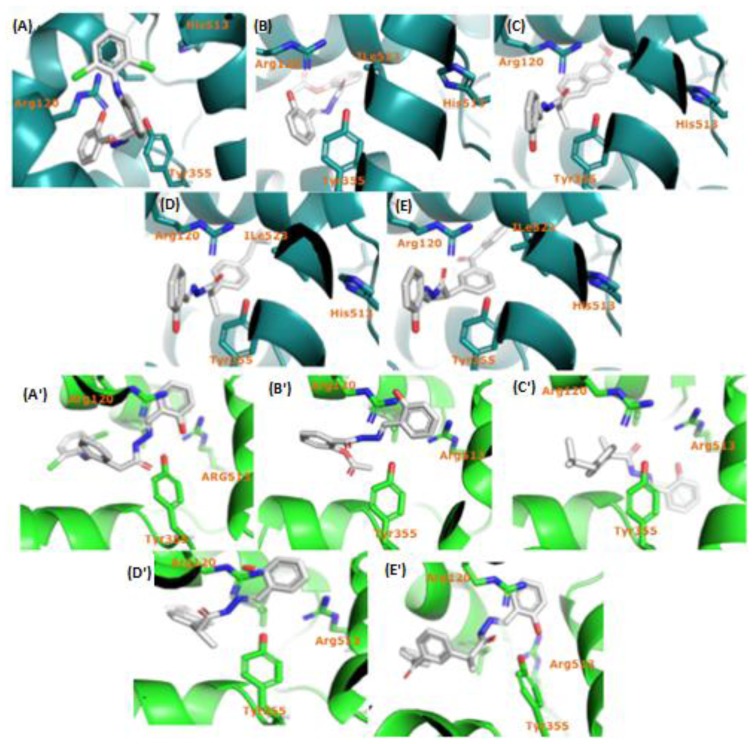
Results docking synthesized analogs: COX-1 blue (**A**–**E**) and COX-2 green (**A′**–**E′**). (**A**) Docking (**4a**); (**B**) Docking (**4b**); (**C**) Docking (**4c**); (**D**) Docking (**4d**) and (**E**) Docking (**4e**) with COX-1 enzyme. (**A′**) Docking (**4a**); (**B′**) Docking (**4b**); (**C′**) Docking (**4c**); (**D′**) Docking (**4d**) and (**E′**) Docking (**4e**) with COX-2 enzyme.

For compounds **4a**–**e**, we performed 100 successive docking interactions with optimization of the interaction to the molecular geometry of the prior interaction. The best-ranked scoring configuration of each ligand-enzyme complex was selected based on its energetic properties. The results were analyzed separately for the NAH derivatives **4a**–**e** and both COX isoforms. The results are shown in Figure 4, for COX-1 in blue and COX-2 in green.

The interaction studies revealed that Ile523 is involved in the interactions between compounds **4a**–**e** and COX-1 (Figure 4A–E). The positions of compounds **4c**–**e** were similar to the cocrystallized structures of COX-1 with either salicylic acid [36] or ibuprofen [37], and this indicates that the compounds favor ionic-type interactions and form hydrogen bonds with residues Arg120 and Tyr355. Compounds **4a** and **4b** were positioned in an inverted position compared with the reference structures (Figure 4A′–E′). According to the best-ranked positions, compounds **4a**–**e** were in close proximity to the hydrogen donor groups of residues Arg120 and Tyr355, which suggests these compounds show higher affinity for COX-2 than for COX-1.

## 3. Experimental Section

### 3.1. General Procedures

Infrared spectra (IR) (KBr disc) were produced on a Shimadzu FTIR-8300 (Shimadzu, Tokyo, Japan) and the frequencies were expressed in cm^−1^. Hydrogen-1 nuclear magnetic resonance (^1^H-NMR) spectra were recorded on a Bruker DRX-400 (300 MHz) (Bruker Biospin Gmbh, Rheinstetten, Germany). All ^1^H-NMR scan were obtained using deuterated dimethyl sulfoxide (DMSO-*d*_6_) as the solvent. Chemical shifts were expressed in parts per million (ppm) relative to tetramethylsilane. Splitting patterns are as follows: s, singlet; d, doublet; dd, double doublet; dt, double triplet; m, multiplet; br, broad. Elemental analysis (C, H, and N) were obtained on a Perkin Elmer model 240C analyzer (Perkin Elmer Inc., Waltham, MA, USA), and the data were within ±0.4% of the theoretical values. Mass spectra were recorded on a JEOL 5 × 10^2^/DA-6000 Mass spectrometer/Data System (JEOL Inc., Peabody, MA, USA) using Argon/Xenon (White Martins, São Paulo, Brazil) (6 kV, 10 mA) as the FAB gas. High-performance liquid chromatography (HPLC) was performed on a Shimadzu (Tokyo, Japan) LC-10AD chromatograph equipped with a model SPD-10A ultraviolet-visible (UV/Vis) detector (Shimadzu, Tokyo, Japan). All compounds were analyzed via HPLC, and their purity was confirmed to be greater than 98.5%. The compounds were separated on a reversed phase C18 column (5 μm particle, 250 mm × 4.6 mm I.D.) Shimadzu (Tokyo, Japan) Shim-pack CLC-ODS (M). HPLC-grade solvents (acetonitrile, methanol and acetic acid) were used in the analyses and were bought from Sigma-Aldrich (St. Louis, MO, USA). The progress of all reactions was monitored by thin layer chromatography (TLC), which was performed on 2.0 × 6.0 cm^2^ aluminum sheets precoated with silica gel 60 (HF-254, Sigma-Aldrich, St. Louis, MO, USA) to a thickness of 0.25 mm. The developed chromatograms were viewed under UV light (254–265 nm) and treated with iodine vapor. Sigma-Aldrich (St. Louis, MO, USA) silica gel (70–230 mesh) was used for preparative column chromatography. Reagents and solvents were purchased from commercial suppliers and used as received.

### 3.2. Preparation

The ester (**5a**–**e**) and hydrazide (**6a**–**e**) derivatives were prepared according to procedures previously described in the literature [2,3,14,20]. The compound **4a**–**b** was prepared according to methods previously described in the literature [43–46].

#### 3.2.1. General Procedures for the Preparation of Ester (**5a**–**e**) and Hydrazide (**6a**–**e**) Derivatives

The carboxylic acid function of non-steroidal anti-inflammatory (diclofenac, salicylic acid, naproxen, ibuprofen or ketoprofen) (0.01 mol) in methanol (15 mL) was reacted under acid catalysis (glacial acetic acid) at 45–50 °C for 30 h. The solvent was evaporated under reduced pressure to form methyl esters **5a**–**e** in good yields (95%–99%). A solution of 0.01 mol of the appropriate methyl ester derivatives (**5a**–**e**) and 5 mL of 80% hydrazine hydrate in 10 mL of methanol was stirred at room temperature for 24 h. The optimal reaction time was determined after TLC monitoring. Then, the mixture was allowed to cool and was neutralized with chloridric acid 37%, resulting in the formation of a white precipitate, which was filtered, washed with water, and dried under reduced pressure to give hydrazide derivatives (**6a**–**e**) in good yields (90%–95%).

#### 3.2.2. General Procedures for the Preparation of *N*-Acyl Hydrazone Derivatives (**4a**–**e**)

Aldehyde salicylic (0.01 mol) was added to a solution of hydrazide derivatives (**6a**–**e**) (0.01 mol) in 15 mL of ethanol in the presence of catalytic amount of hydrochloric acid. The reaction was stirred for 1–3 h at room temperature and monitored by TLC. Subsequently, the solvent was evaporated under reduced pressure. The precipitate was collected by filtration, washed with cold water, and dried under vacuum to give the *N-*acyl hydrazone derivatives (**4a**–**e**). In addition, the compounds were purified by column chromatography (flash silica, eluent: 60% ethyl acetate; 40% hexane) with good yields.

(*E*)-2-(2-((2,6-Dichlorophenyl)amino)phenyl)-*N′*-(2-hydroxybenzylidene)acetohydrazide (**4a**). This compound was obtained according to adapted methodology [43–45]. Derivative **4b** was obtained as a white solid by condensation of **6a** with salicylaldehyde with 75% yield. ^1^H-NMR (DMSO-*d*_6_) δ: 3.79 (s, 2H, C*H*_2_COOH), 6.26 (d, 1H), 6.85 (t, 1H), 6.97 (t, 1H), 7.03–7.07 (m, 3H), 7.19–7.23 (m, 2H), 7.44 (t, 1H), 7.52–7.54 (m, 2H), 8.52 (s, 1H, C*H*=N), 9.01 (s, 2H, N–*H*), 11.13 (s, 1H, O–*H*) ppm; IR (cm^−1^): 3370 (N–H_ax_), 3331 (O–H_ax_), 3028, 2971, 2922 (C–H_sim_), 1647 (CH=N), 1451 (C_Ar_–H). MS-EI: 413.07 (*m*/*z*). Calculated for C_21_H_17_Cl_2_N_3_O_2_: C, 60.88; H, 4.14; N, 10.14. Found: C, 60.49; H, 4.41; N, 10.55.

(*E*)-2-Hydroxy-*N′*-(2-hydroxybenzylidene)benzohydrazide (**4b**). This compound was obtained according to adapted methodology [43–45]. Derivative **4b** was obtained as a white solid by condensation of **6b** with salicylaldehyde with 82% yield. ^1^H-NMR (DMSO-*d*_6_) δ: 6.92–7.00 (m, 4H), 7.32 (t, 1H, *J*_o_ = 7.4 Hz), 7.46 (t, 1H, *J*_o_ = 7.2 Hz), 7.57 (d, 1H, *J*_o_= 7.4 Hz), 7.9 (d, 1H, *J*_o_= 7.2 Hz), 8.68 (s, 1H, C*H*=N), 11.21 (s, 1H, N–*H*), 11.98 (s, 2H, O–*H*) ppm; IR (cm^−1^): 3343 and 3279 (N–H_ax_), 3330 (O–H_ax_), 3030, 2975, 2926 (C–H_sim_), 1641 (CH=N), 1450 (C_Ar_–H). MS-EI: 256.08 (*m*/*z*). Calculated for C_14_H_12_N_2_O_3_: C, 65.62; H, 4.72; N, 10.93. Found: C, 65.38; H, 4.61; N, 10.72.

(*E*)-*N′*-(2-Hydroxybenzylidene)-2-(6-methoxynaphthalen-2-yl)propanehydrazide (**4c**): Derivative **4c** was obtained as a yellow solid by condensation of **6c** with salicylaldehyde with 78% of yield. ^1^H-NMR (DMSO-*d*_6_) δ: 1.50 (d, 3H, C*H*_3_), 3.84 (q, 1H, C–*H*–CH_3_), 3.87 (s, 3H, OC*H*_3_), 6.83–6.90 (m, 2H), 7.24–7.29 (m, 2H), 7.45–7.51 (m, 2H), 7.66–7.73 (m, 1H), 7.77–7.82 (m, 3H), 8.40 (s, 1H, C*H*=N), 11.20 (s, 1H, N–*H*), 11.81 (s, 1H, O–*H*) ppm; IR (cm^−1^): 3339 (N–H_ax_), 3333 (O–H_ax_), 3030, 2976, 2922 (C–H_sim_), 1640 (CH=N), 1451 (C_Ar_–H). MS-EI: 348.15 (*m*/*z*). Calculated for C_21_H_20_N_2_O_3_: C, 72.40; H, 5.79; N, 8.04. Found: C, 72.22; H, 5.47; N, 7.82.

(*E*)-*N′*-(2-Hydroxybenzylidene)-2-(4-isobutylphenyl)propanehydrazide (**4d**): Derivative **4d** was obtained as a yellow-white solid by condensation of **6d** with salicylaldehyde with 65% yield. ^1^H-NMR (DMSO-*d*_6_) δ: 0.85 (d, 6H, C*H*_3_), 1.40 (d, 3H, C*H*_3_), 1.80 (m, 1H, C–*H*), 2.41 (d, 2H, –C*H*_2_–), 3.66 (q, 1H, C–*H*–CH_3_), 6.89 (d, 1H), 7.08–7.12 (m, 2H), 7.21–7.29 (m, 4H), 7.46–7.49 (d, 1H), 8.38 (s, 1H, C*H*=N), 11.21 (s, 1H, N–*H*), 11.79 (s, 1H, O–*H*) ppm; IR (cm^−1^): 3342 (N–H_ax_), 3335 (O–H_ax_), 3031, 2974, 2920 (C–H_sim_), 1642 (CH=N), 1438 (C_Ar_–H). MS-EI: 324.18 (*m*/*z*). Calculated for C_20_H_24_N_2_O_2_: C, 74.04; H, 7.46; N, 8.64. Found: C, 74.41; H, 7.22; N, 8.60.

(*E*)-2-(3-Benzoylphenyl)-*N′*-(2-hydroxybenzylidene)propanehydrazide (**4e**): Derivative **4e** was obtained as a yellow-white solid by condensation of **6d** with salicylaldehyde with 61% yield. ^1^H-NMR (DMSO-*d*_6_) δ: 1.33 (d, 3H, C*H*_3_), 3.62 (q, 1H, C–*H*–CH_3_), 6.89 (d, 1H), 7.07–7.12 (m, 2H), 7.46–7.49 (d, 1H), 7.55–7.67 (m, 10H), 8.38 (s, 1H, C*H*=N), 11.19 (s, 1H, N-*H*), 11.83 (s, 1H, O–*H*) ppm; IR (cm^−1^): 3339 (N–H_ax_), 3328 (O–H_ax_), 3030, 2973, 2921 (C–H_sim_), 1644 (CH=N), 1436 (C_Ar_–H). MS-EI: 372.15 (*m*/*z*). Calculated for C_23_H_20_N_2_O_3_: C, 74.18; H, 5.41; N, 7.52. Found: C, 74.31; H, 5.32; N, 7.53.

### 3.3. Pharmacology

Adult male Wistar rats (150–200 g) and Swiss albino mice (20–35 g) were used in the experiments. They were housed in single-sex cages under a 12 h light:12 h dark cycle (lights on at 6 am) in a controlled-temperature room (22 ± 2 °C). The rats and mice had free access to food and water. The experiments were performed after the ethical protocol was approved by the local Institutional Ethics Committee. All experiments were performed in accordance with the current guidelines for the care of laboratory animals and the ethical guidelines for the investigation of experimental pain in conscious animals [47].

#### 3.3.1. Anti-Inflammatory Activity

The anti-inflammatory activity was evaluated using the carrageenan-induced rat paw edema method [23]. Wistar rats were divided into groups of six animals each. Animals of the control group were treated with an aqueous solution of carboxymethyl cellulose (CMC, 0.5% *w*/*v*). All compounds (**4a**–**e**), parental drugs, and indomethacin were administered by oral route using gavage at 300 μmol kg^−1^ in a homogeneous suspension of sodium carboxymethyl-cellulose (0.5% *w*/*v*). Each animal received by oral administration using gavage 0.75–1.0 mL of the respective drugs. Thirty minutes after the administration of the drugs, each rat received a subplantar injection of 0.1 mL of a freshly prepared 1% carrageenan solution in its left hind paw. The measurement of the hind paw was carried out using a thickness gauge before each treatment (*V*_o_) and in each interval (*V*_t_) after the administration of drugs. The percentage of swelling inhibition was calculated using the following equation: Inhibition (%) = {[(*V*_t_ − *V*_o_)_control_ − ((*V*_t_ − *V*_o_)_treated_]/(*V*_t_ − *V*_o_)_control_} × 100, where *V*_t_ and *V*_o_ relate to the average volume in the hind paw of the rats after given the compounds orally (treatment group) and before any treatment. All the results are expressed as percentage of inhibition mean ± S.E.M. The data were analyzed statistically using ANOVA and Tukey’s Multiple Comparison Test at significance level of *p* < 0.01.

#### 3.3.2. Analgesic Activity

Analgesic activity was determined *in vivo* with the acetic-acid-induced (1% (*v*/*v*) acetic acid solution) abdominal constriction test in Swiss mice (25–30 g) [25]. The compounds (**4a**–**e**) and parental NSAIDs were administered orally (100 μmol/kg) as a suspension in carboxymethyl cellulose (CMC, 0.5% *w*/*v*) (vehicle). Dipyrone (100 μmol/kg) was used as the standard drug, administered under the same conditions. Acetic acid solution was administered i.p. 1 h after the administration of the compounds. Ten minutes after the i.p. acetic acid injection, the number of constrictions per animal was recorded for 20 min. The control animals received an equal volume of vehicle. Antinociceptive activity was expressed as percentage inhibition of the constrictions compared with those in the vehicle-treated control group. The results are expressed as means ± S.E.M. of 8 animals per group. The data were analyzed statistically using ANOVA and Tukey’s Multiple Comparison Test at significance level of *p* < 0.01.

#### 3.3.3. Ulcerogenicity Studies

Gastrointestinal toxicity was determined using the method previously described [6,27,28]. The studies were carried out on healthy Wistar rats (150–200 g) at 300 μmol·kg^−1^. The animals were divided into groups of six animals each. Group 1 was used as negative control and received vehicle only, group 2 and 3 received pure diclofenac and celecoxib at 300 μmol·kg^−1^ respectively. The groups 4–8 received compounds **4a**–**e** at 300 μmol·kg^−1^. The animals were fasted 8 h prior to receiving the treatments. Subsequently, they received free access to food and water and were sacrificed 17 h later. The quantification of ulcers was done in a blinded fashion with one researcher counting ulcers not knowing to which experimental group the animal belonged. The rats’ gastric mucosa was examined using a 4× binocular magnifier (Nikkon, Melville, NY, USA). The lesions were counted and divided into large (larger than 2 mm in diameter), small (1–2 mm), and punctiform (less than 1 mm). For each stomach, the severity of mucosal damage was assessed according to the following scoring system: 0—no lesions or up to five punctiform lesions; 1—more than five punctiform lesions; 2—one to five small ulcers; 3—more than five small ulcers or one large ulcer; 4—more than one large ulcer. The mean score of each treated group minus the mean score of the control group was considered as the “severity index” of gastric damage. Statistical analysis was performed with ANOVA followed by Tukey’s Multiple Comparison Test. * *p* < 0.05.

### 3.4. Docking Studies

Docking studies of the compounds were performed using crystal structures of COX-1 (PDB ID: 1PTH) and COX-2 (PDB ID: 1CX2) enzymes sequences obtained from the Protein Data Bank (http://www.rcsb.org/pdb/home/home.do). All the docking simulations were carried out using the program GOLD (GOLD-Protein Ligand Docking) version 5.1, (Cambridge Crystallographic Data Center—CCDC Inc., Cambridge, UK). The protein molecule was prepared using the Biopolymer module implemented in SYBYL 8.0 (Certara, St. Louis, MO, USA). The ligand and water molecules were removed from the binding pocket and hydrogen atoms were added in standard geometry [47]. Three-dimensional structures of the aforementioned compounds were constructed using Chem 3D ultra 13.0 software (Chemical Structure Drawing Standard; Perkin Elmer Informatics, Waltham, MA, USA). Compound structures were optimized in SPARTAN 010′ program (Wavefunction Inc., Irvine, CA, USA) by RM1 method application, using internal default settings for convergence criteria. The center of the grid docking box was determined by coordinates *X*, *Y* and *Z* of carbon four of the Try 355 aromatic ring; for 1PTH these were 24.992, 33.178 and 217.594 and for 1CX2 these were 23.715, 20.311 and 23.677. The compounds were subjected to flexible docking using the pre-computed grid files. For each of the 20 independent runs were performed to evaluate the stability of the docking results. Standard set parameters were used in all the calculations. The 100 top-scored poses were saved and analyzed and only the best scoring poses were selected for the study.

## 4. Conclusions

We synthesized a series of NSAID hybrids containing a NAH subunit (compounds **4a**–**e**) with yields of 61%–82%. All five compounds were characterized by ^1^H-NMR spectroscopy, infrared spectroscopy, mass spectrometry, and elemental analysis. Molecular docking studies suggested that compounds **4a**–**e** bind to both COX isoforms, but with higher affinity for COX-2. The *in vivo* anti-inflammatory activities of compounds **4a**–**e** were generally similar, although compound **4c** showed the greatest anti-inflammatory activity in terms of inhibiting rat paw edema, with inhibitory rates of 35.9% and 52.8% at 2 and 4 h, respectively. The anti-inflammatory activity of NAH derivatives was inferior to their respective parent drugs, except for compound **4c** after 5 h. Compounds **4a**–**e** were also less gastrotoxic than the parent drug. In addition, compounds **4b**–**e** induced mucosal damage comparable to celecoxib and the majority of ulcers induced by compounds **4b**–**e** were punctiform (<1 mm). The *in vivo* analgesic activities of the compounds were higher than the respective parent drug for compounds **4a**–**b** and **4d**–**e**. Compound **4a** was more active than dipyrone in reducing acetic-acid-induced abdominal constrictions. Therefore, the presence of an NAH subunit reduced ulcer formation, but did not increase the efficacy of the hybrid compounds relative to their parent drugs. Taken together, our results suggest that compounds **4a**–**e** are candidate anti-inflammatory and analgesic drugs with reduced gastrotoxic effects, and might be useful for long-term therapy.

## Figures and Tables

**Table 1.** Effect of *N-*acyl hydrazone derivatives **4a**–**e**, parental drugs (diclofenac, salicic acid, naproxen, ibuprofen, ketoprofen), and indomethacin (300 μmol/kg, p.o.) in carrageenan-induced rat paw edema assay.

Compounds	*N*	% Inhibition mean ± S.E.M.					

		60 min	120 min	180 min	240 min	300 min	360 min
Indomethacin	6	66.7 ± 1.3	71.8 ± 0.96	75.7 ± 1.32	78.8 ± 2.13	76.6 ± 1.93	75.7 ± 0.98
Diclofenac	6	59.3 ± 0.27 *	64.7 ± 1.21 *	66.6 ± 1.48 *	69.2 ± 1.71 *	68.2 ± 0.98 *	75.1 ± 2.11 *
**4a**	6	0 ^†^	0 ^†^	0 ^†^	0 ^†^	0 ^†^	0 ^†^
Salicylic acid	6	63.4 ± 2.31 *	58.9 ± 1.83 *	73.3 ± 2.44 *	73.0 ± 1.17 *	72.7 ± 1.76 *	74.1 ± 2.10 *
**4b**	6	7.6 ± 0.10 ^†^	14.9 ± 0.08 ^†^	11.3 ± 0.07 ^†^	12.5 ± 0.09 ^†^	12.2 ± 0.08 ^†^	32.1 ± 0.05 ^†^
Naproxen	6	65.8 ± 1.32 *	72.7 ± 1.32 *	69.2 ± 1.61 *	58.8 ± 0.91	53.9 ± 1.77	41.9 ± 1.87
**4c**	6	8.0 ± 0.10 ^†^	35.9 ± 0.11 ^†^	35.0 ± 0.11 ^†^	52.8 ± 0.07 ^†^	67.5 ± 0.04 ^†,^*	81.5 ± 0.04 *
Ibuprofen	6	64.8 ± 1.86 *	42.1 ± 2.18 *	46.7 ± 1.73 *	42.3 ± 1.6 *	45.5 ± 1.55 *	62.5 ± 2.09 *
**4d**	6	22.7 ± 0.07 ^†^	25.7 ± 0.05 ^†^	9.7 ± 0.09 ^†^	10.6 ± 0.08 ^†^	22.9 ± 0.08 ^†^	45 ± 0.07 ^†^
Ketoprofen	6	71.8 ± 1.68 *	64.7 ± 1.54 *	63.8 ± 2.21 *	66.9 ± 1.72 *	72.7 ± 1.31 *	75.1 ± 1.84 *
**4e**	6	11.3 ± 0.08 ^†^	15.7 ± 0.04 ^†^	16.1 ± 0.05 ^†^	18.5 ± 0.07 ^†^	29.4 ± 0.1 ^†^	34.7 ± 0.12 ^†^

Data are expressed as means ± S.E.M. Statistical differences between the treated and the control groups were evaluated by ANOVA and Tukey’s Multiple Comparison Test (*p* < 0.01). The asterisks (*) denote the levels of significance between the parent drug and **4a**–**e**; The cross marks (^†^) denote the level of significance between the indomethacin group and compounds **4a**–**e**.

**Table 2.** Antinociceptive effect of dypirone, compounds (**4a**–**e**), and parent NSAIDs using acetic acid-induced abdominal constrictions.

Compounds	% Protection
Dypirone	37.2 ± 1.4
Diclofenac	28.3 ± 2.2
**4a**	42.0 ± 1.1 ^†,^*
Salicylic acid	30.2 ± 1.5
**4b**	36.8 ± 2.1 *
Naproxen	36.8 ± 0.8
**4c**	36.4 ± 1.7
Ibuprofen	25.0 ± 0.7
**4d**	28.1 ± 1.0 ^†^
Ketoprofen	22.3 ± 0.9
**4e**	25.6 ± 2.2 ^†^

Data are expressed as the inhibition percentage of total writhings calculated from eight animals. Statistical differences between the treated and the control groups were evaluated by ANOVA and Tukey’s Multiple Comparison Test (*p* < 0.01). The asterisks (*) denote the levels of significance between the parent NSAIDs and compounds **4a**–**e**; The cross marks (^†^) denote the level of significance between the standard dypirone group and compounds **4a**–**e**.

**Table 3.** Ulcerogenic effect of diclofenac and compounds **4a**–**e** in rats (*n* = 6, mean ± S.D.).

Compounds	Number of ulcers	<1 mm	1–2 mm	>2 mm
Celecoxib	11 ± 3.1	7.5 ± 2.7 (68.2%)	3.5 ± 1.4 (31.8%)	-
Diclofenac	72 ± 6.9	60 ± 7.7 (83.3%)	8.1 ± 5.3 (11.3%)	3.9 ± 3.1 (5.4%)
**4a**	26 ± 7.7 ^†,^*	20.8 ± 4.8 (80%)	5.2 ± 2.1 (20%)	-
Salicylic acid	69.8 ± 3.4	52.56 ± 5.2 (75.2%)	9.21 ± 3.6 (13.2%)	8.03 ± 2.7 (11.6%)
**4b**	17.5 ± 3.3 *	14.3 ± 3.9 (81.7%)	3.2 ± 1.1 (18.3%)	-
Naproxen	57.8 ± 4.2	47.97 ± 2.7 (83%)	9.82 ± 0.8 (17%)	-
**4c**	7.1 ± 2.8 *	6.5 ± 2.8 (91.5%)	0.6 ± 0.2 (8.5%)	-
Ibuprofen	70.7 ± 4.4	52.31 ± 6.1 (73.9)	16.54 ± 2.8 (23.4%)	1.83 ± 0.8 (2.7%)
**4d**	14.1 ± 3.5 *	13 ± 3.6 (92.2%)	1.1 ± 1.2 (7.8%)	-
Ketoprofen	68.9 ± 3.3	59.46 (86.3%)	8.33 ± 1.7 (12.1%)	1.10 ± 0.4 (1.6%)
**4e**	8.2 ± 2.2 *	7.4 ± 2.9 (90.2%)	0.8 ± 0.4 (9.8%)	-

Statistical differences between the treated and the control groups were evaluated by ANOVA and Tukey’s Multiple Comparison Test (*p* < 0.05). The asterisks (*) denote the levels of significance between the parent drug and the respective *N*-acyl derivative (**4a**–**e)**; The cross mark (^†^) denotes the level of significance between the standard celecoxib group and the respective *N*-acyl derivative (**4a**–**e**).
